# Characterization of Resistance Patterns and Detection of Apramycin Resistance Genes in *Escherichia coli* Isolated from Chicken Feces and Houseflies after Apramycin Administration

**DOI:** 10.3389/fmicb.2018.00328

**Published:** 2018-02-27

**Authors:** Anyun Zhang, Yunxia Li, Zhongbin Guan, Hongmei Tuo, Dan Liu, Yanxian Yang, Changwen Xu, Changwei Lei, Hongning Wang

**Affiliations:** ^1^Key Laboratory of Bio-resource and Eco-environment, Ministry of Education, College of Life Sciences, Sichuan University, Chengdu, China; ^2^Animal Disease Prevention and Food Safety Key Laboratory of Sichuan Province, Chengdu, China

**Keywords:** apramycin resistance genes, *Escherichia coli*, PFGE, chicken feces, housefly

## Abstract

The aim of this study was to evaluate the influence of apramycin administration on the development of antibiotic resistance in *Escherichia coli* (*E. coli*) strains isolated from chicken feces and houseflies under field conditions. Chickens in the medicated group (*n* = 25,000) were given successive prophylactic doses (0.5 mg/l) of apramycin in their drinking water from Days 1 to 5, while no antibiotics were added to the un-medicated groups drinking water (*n* = 25,000). Over 40 days, a total of 1170 *E. coli* strains were isolated from fecal samples obtained from medicated and un-medicated chickens and houseflies from the same chicken farm. Apramycin MIC90 values for *E. coli* strains obtained from the medicated group increased 32–128 times from Days 2 to 6 (256–1024 μg/ml) when compared to those on Day 0 (8 μg/ml). Strains isolated from un-medicated chickens and houseflies had consistently low MIC90 values (8–16 μg/ml) during the first week, but showed a dramatic increase from Days 8 to 10 (128–1024 μg/ml). The apramycin resistance gene *aac(3)-IV* was detected in *E. coli* strains from medicated (*n* = 71), un-medicated (*n* = 32), and housefly groups (*n* = 42). All strains positive for *aac(3)-IV* were classified into 12 pulsed-field gel electrophoresis (PFGE) types. PFGE types A, E, and G were the predominant types in both the medicated and housefly groups, suggesting houseflies play an important role in spreading *E. coli*-resistant strains. Taken together, our study revealed that apramycin administration could facilitate the occurrence of apramycin-resistant *E. coli* and the apramycin resistance gene *acc(3)-IV*. In turn, these strains could be transmitted by houseflies, thus increasing the potential risk of spreading multi-drug-resistant *E. coli* to the public.

## Introduction

Antimicrobial resistance emerges from the use of antimicrobials in animals and the subsequent transfer of resistance bacteria from those animals to the broader environment (Berendonk et al., 2015). The influence of antimicrobial usage on the prevalence of resistant strains in animals is of great concern for wider public health (da Costa et al., 2008; Martins da Costa et al., 2011; Sato et al., 2014).

Apramycin is an aminoglycoside antibiotic that has been used in animal husbandry since the early 1980s. It is still used in several European countries and it was approved for use in China in 1999 (Zhang et al., 2009). It is used to treat or prevent infections caused by Gram-negative bacteria such as colibacillosis, salmonellosis, and bacterial enteritis in poultry, swine, and calves (Antunes et al., 2011). Epidemiological investigations of apramycin-resistant bacteria from food producing animals showed differential prevalence of apramycin resistance in different animals (Choi et al., 2011). To date, there are two known resistance genes that confer resistance to apramycin in *E. coli*. One is the most prevalent apramycin resistance gene, *aac(3)-IV*, which codes for an aminoglycoside 3-*N*-acetyltransferase type-IV enzyme (Davies and Oconnor, 1978). The other is *npmA*, which was identified in a clinical *E. coli* strain in 2007 and subsequently found to encode for a 16S rRNA m^1^A1408 methyltransferase (Wachino et al., 2007).

According to a previous study in China, apramycin-resistant *E. coli* are not only resistant to apramycin itself, such strains have also been found to be multi-resistant to several other antimicrobial agents (Zhang et al., 2009). This could complicate therapeutic options for bacteriosis treatment in both farm animals and humans (Zhang et al., 2009). A few studies have shown that apramycin treatment caused significant selective pressure in prevalence of resistance *E. coli* in swine (Mathew et al., 2003; Jensen et al., 2006). However, its influence on *E. coli* found in chicken has not yet been investigated.

The risk of flies disseminating resistant bacteria from livestock and poultry farms to the public has been a subject of increasing concern. Flies captured from different animal rearing facilities had been shown to be vectors for different microorganisms, some of which may be foodborne pathogens that are potentially threatening to human health (Forster et al., 2007). Moreover, flies also function as transmission vehicles for ESBL-producing *E. coli* from cattle (Usui et al., 2013) as well as laying hens and broilers (Blaak et al., 2014). However, the influence of apramycin administration on the development of antibiotic resistance in *E. coli* from chicken feces and houseflies has not been fully investigated.

Given this, our study was designed to evaluate three questions: (i) the influence on the development and persistence of apramycin resistance in *E. coli* isolated from fecal and houseflies in a chicken farm after preventive use of apramycin; (ii) the relationships between apramycin-resistant *E. coil* isolated from chicken feces and houseflies; and (iii) the characterization of apramycin-resistant *E. coli* found in houseflies.

## Materials and Methods

### Study Setting

This study was conducted in a chicken farm with two different poultry houses (1000 m^2^ each). The two houses were separated about 50 m to each other. After hatching, 50,000 chickens were equally and randomly allocated into two poultry houses (Day 0). Chickens in the medicated group (*n* = 25,000) were given successive prophylactic doses (0.5 mg/l) of apramycinsulfate (Shandong Qilu King-phar Pharmaceutical Co., Ltd., Shandong, China) in their drinking water from Days 1 to 5. In comparison, the un-medicated group (*n* = 25,000) was given drinking water without apramycin. No other antibiotics were used during the study period. Add antibiotic to drinking water for 5 days is the normal production behavior of the laying hens company. This study was carried out without any additional interference with the growth of the chickens. The protocol was approved by the Animal Ethics Committee of Sichuan University. We confirm that the best practice veterinary care and informed consent has been granted by the owners.

Samples were taken from each group as described in **Table 1**. Specifically, 15 cloacal swabs were collected from both the medicated and un-medicated groups at Day 0 and placed separately into sterile plastic bags. Fifteen sterilized plates were randomly placed under selected cages along two main diagonals of the poultry house containing both the medicated and un-medicated groups. Plates were placed at 12:00 am and withdrawn at 3:00 pm to allow for the collection of fresh fecal samples. Collections occurred on Days 1, 2, 3, 4, 5, 6, 8, 10, 15, 20, 30, and 40. Flies were captured using a sweep net on each sampling day from both of the two houses and approximately 30 flies were individually placed into sterile tubes for later morphological classification. All samples were placed into cool boxes containing ice packs and transported to the lab within 4 h for immediate bacterial isolation.

**Table 1:** Sample collection and *E. coli* isolation.

Groups	Sample types	Number of samples/number of *E. coli* isolated			Total number of *E. coli*
		Pre-medication^a^	On-medication^b^	Off-medication^c^	
Un-medicated group	Cloacal swab	15/30	–	–	390
	Fresh feces	–	15/30	15/30	
Medicated group	Cloacal swab	15/30	–	–	390
	Fresh feces	–	15/30	15/30	
Houseflies group	Housefly	15/30	15/30	15/30	390
Total number of *E. coli*		90	450	630	1170

*^a^Sampling time at day 0 when chicken was hatched and transferred to the farm. ^b^Sampling time at Days 1–5 when apramycin was administrated. ^c^Sampling time at Days 6, 8, 10, 15, 20, 30, and 40 after apramycin was administrated.*

### Bacterial Isolation

The cloacal swabs (*n* = 30) were separately put into 10 ml phosphate-buffered saline (PBS) and thoroughly vortexed. The resulting suspension was then 10-fold serial diluted with PBS and 100 μl of the dilution was plated onto eosin methylene blue (EMB) agar (Hangzhou Microbial Reagent Co., Ltd., Hangzhou, China) and incubated at 37°C overnight.

Fecal samples were collected from medicated (*n* = 15) and un-medicated groups (*n* = 15) at each sampling time. From these fresh fecal samples, 0.1 g was put into 10 ml PBS and thoroughly vortexed. The resulting suspension was 10-fold serial diluted with PBS and 100 μl was plated onto EMB agar and incubated at 37°C overnight.

Houseflies were collected at each sampling time, as previously described. Collected houseflies were morphologically identified using a stereomicroscope and 15 houseflies were randomly chosen for subsequent *E. coli* isolation. Each housefly was put into 10 ml PBS and thoroughly vortexed. The resulting suspension was 10 times gradient diluted with PBS, 100 μl was plated onto EMB agar, then incubated at 37°C overnight.

After overnight incubation, two colonies from each plate were selected for each sample. All isolates were then confirmed as being *E. coli* using a biochemical identification kit for *Enterobacteriaceae* (Hangzhou Microbial Reagent Co. Ltd., Hangzhou, China). All the confirmed *E. coli* isolates were kept frozen (-70°C) with 25% glycerol pending further analysis.

### Antimicrobial Susceptibility Testing

The minimum inhibitory concentration (MIC) of apramycinsulfate (China Institute of Veterinary Drugs Control, Beijing, China) for all *E. coli* isolates was determined using the agar dilution method following the guidelines of the Clinical and Laboratory Standards Institute [CLSI] (2012a). In short, *E. coli* strains were subcultured on Luria Bertani (LB) agar at 37°C for 12 h. A clearly separate colony of the *E. coli* isolate was picked and a suspension of each strain in saline solution was adjusted to match the 0.5 McFarland standard. Mueller–Hinton (MH) plates that contain different apramycinsulfate concentration (0.125–1024 μg/ml) were seeded with a multipoint inoculum replicator and incubated at 35°C for 16–18 h. *E. coli* ATCC 25922 was used as the quality control strain. MIC data were only accepted if MICs of the control strains were within the required reference ranges. MIC90 (the MIC that ≥90% tested bacteria were inhibited for each sampling group) was used to evaluate the changes trend of apramycin resistance.

### Apramycin Resistance Gene Detection

For detection of apramycin resistance genes, genomic DNA was prepared using a QIAamp DNA Mini Kit according to the manufacturer’s instructions (Qiagen Inc., Valencia, CA, United States). Apramycin resistance genes *aac(3)-IV* and *npmA* were screened for all *E. coli* isolates as previously described (Yates et al., 2004; Zhou et al., 2010).

### Pulsed-Field Gel Electrophoresis (PFGE) Typing of *aac(3)-IV-*Positive Strains

The clonal relatedness of *aac(3)-IV*-positive isolates were typed by PFGE as previously described (Gautom, 1997). Briefly, 145 *aac(3)-IV*-positive isolates were subcultured on LB agar at 37°C for 12 h. A single colony of each isolate was suspended with cotton swab in about 2 ml of TE buffer. The cell suspensions were adjusted to 20% transmittance by using a bioMérieux Vitek (Hazelwood, MO, United States). Proteinase K and lysozyme were added into 100 ml cell suspensions at final concentration of 1 mg/ml each and then incubated at 37°C for 10–15 min. Following the lysozyme–proteinase K incubation, 7 ml of 20% sodium dodecyl sulfate (50°C) and 140 ml of 1.2% InCert Agarose (50°C) were mixed with each bacterial suspension. Then the mixture was immediately added to plug molds (Bio-Rad Laboratories). After that, each solid plug was transferred to 2-ml round-bottom tubes with 1.5 ml of ESP buffer and incubated at 55°C for 2 h in a water bath. Then five times washes with 8–10 ml TE buffer (50°C) each in a shaker water bath for 15 min were carried out. For restriction endonuclease digestion, two 1-mm-thick slices of each plug were incubated at 37°C for 3 h with 50 U of *Xba*I enzyme. The plugs were then soaked in standard 0.5 Tris–borate–EDTA (TBE) prior to electrophoresis. The electrophoretic conditions used were as follows: initial switch time, 2.16 s; final switch time, 54.17 s; run time, 22 h; angle, 120°; gradient, 6.0 V/cm; temperature, 14°C; ramping factor, linear. PFGE profiles were analyzed using the BioNumerics Program (Applied Maths, Sint-Martens-Latem, Belgium) as previously described (Yates et al., 2004). The clonal clusters with a similarity cutoff value of 80% were used in this study.

### Antimicrobial Resistance Phenotype and Genotype of *aac(3)-IV*-Positive Strains

To investigate the antimicrobial resistance patterns and resistance genes of *aac(3)-IV*-positive isolates belonging to different PFGE types, we tested one isolate of each PFGE type for susceptibility to 22 antimicrobial agents. This process was conducted using the disk diffusion method according to CLSI guidelines (Clinical and Laboratory Standards Institute [CLSI], 2012b). Briefly, MH agar plate was inoculated with suspensions of bacteria, equivalent to standard 0.5 McFarland. Subsequently, the disks of different antimicrobial agents were placed on media and then incubated at 35°C for 16–18 h. The tested antimicrobial agents were as follows: ampicillin (10 μg), piperacillin (100 μg), cefazolin (30 μg), ceftazidime (30 μg), cefotaxime (30 μg), ceftriaxone (30 μg), cefepime (30 μg), amoxicillin/clavulanic acid (20/10 μg), ampicillin/sulbactam (10/10 μg), piperacillin/tazobactam (100/10 μg), aztreonam (30 μg), imipenem (10 μg), meropenem (10 μg), tetracycline (30 μg), doxycycline (30 μg), ciprofloxacin (5 μg), levofloxacin (5 μg), gentamicin (10 μg), amikacin (30 μg), sulfamethoxazole/trimethoprim (1.25/23.75 μg), chloramphenicol (30 μg), and florfenicol (30 μg). All tested antimicrobial agents were obtained from Oxoid (Basingstoke, United Kingdom). *E. coli* ATCC 25922 was used as the control strain. The obtained data were interpreted according to CLSI recommendations (Clinical and Laboratory Standards Institute [CLSI], 2016).

Finally, we screened for the presence of 25 additional types of resistance genes and integron integrates genes in the 12 *aac(3)-IV*-positive isolates were screened using primers and PCR conditions as previously described: *bla*_TEM_, *bla*_SHV_, *bla*_OXA-1-like_, *bla*_CTX-M-group 1_, *bla*_CTX-M-group 2_, *bla*_CTX-M-group 9_, *bla*_CTX-M-group 8/25_ (Dallenne et al., 2010), *tetA*, *tetB*, *tetM* (Ng et al., 2001), *qnrA*, *qnrB*, *qnrC*, *qnrD* (Schink et al., 2012), *aac(3)-IIa*, *aac(6′)-Ib*, *ant(3″)-Ia*, *aph(3′)-IIa* (Zhang et al., 2012), *sulI*, *sulII* (Kerrn et al., 2002), *cfr*, *cmlA*, *floR* (Keyes et al., 2000; Kehrenberg and Schwarz, 2006), *IntI*, and *IntII* (Ishikawa, 2011).

### Statistical Analysis

Statistical analysis was performed using SPSS software for Windows, version 18.0 (SPSS Inc., Chicago, IL, United States). Data were analyzed using descriptive statistics and χ^2^ tests. A *P*-value < 0.05 was considered statistically significant.

## Results

### Bacterial Isolation

Over the course of the 40-day testing period, a total of 585 samples were collected. Two *E. coli* strains were selected from each sample. As shown in **Table 1**, a total of 1170 *E. coli* isolates from the medicated group (*n* = 390), un-medicated group (*n* = 390), and housefly group (*n* = 390) were obtained. Prior to apramycin administration (Day 0), 90 *E. coli* strains were collected from the included samples, 450 *E. coli* strains were collected during apramycin administration (Days 1–5), and 630 *E. coli* strains were collected after apramycin administration.

### The Changes of MIC90 for Apramycin

**FIGURE 1:**
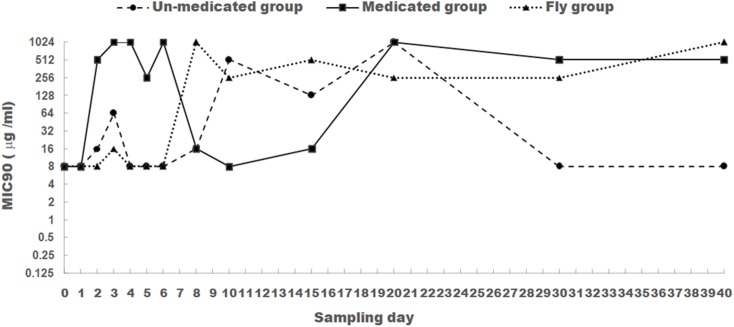
The changes of MIC90 for apramycin of *E. coli* isolated from chicken feces (medicated and un-medicated groups) and houseflies. Apramycin was administrated from Days 1 to 5 in their drinking water (0.5 mg/l) for the medicated group.

Minimum inhibitory concentration for apramycin was tested for all 1170 *E. coli* isolates. MIC90 was used to evaluate the changes trend of apramycin resistance (**Figure 1**).

For *E. coli* isolates obtained from the medicated group, apramycin MIC90 was at a low level (8 μg/ml) prior to apramycin administration (Day 0). After the addition of apramycin, MIC90 increased significantly from Days 2 to 6 and was maintained above 512 μg/ml compared to that in Day 0 and Day 1 (*P* < 0.05). This was with the exception of Day 5, which sustained a level of 256 μg/ml. However, ending apramycin administration resulted in a substantial decrease in MIC90 (8–16 μg/ml) from Days 8 to 15. To our surprise, MIC90 increased again (above 512 μg/ml) from Days 20 to 40.

For *E. coli* isolates obtained from the un-medicated group, apramycin MIC90 was remained at low level (8–16 μg/ml) from Days 0 to 8. This was with the exception of Day 3, which sustained a level of 64 μg/ml. Days 10–20 saw a dramatic increase (128–1024 μg/ml), but a subsequent decrease to 8 μg/ml from Days 30 to 40. Significant difference was found for the MIC90 values between *E. coli* isolates from the un-medicated group and medicated group (*P* < 0.05).

For *E. coli* isolated from houseflies, apramycin MIC90 remained at a low level (8–16 μg/ml) from Days 0 to 6, then increased and fluctuated between 256 and 1024 μg/ml from Days 8 to 40. MIC90 values for apramycin were significantly different between 1–6 days and 8–40 days for *E. coli* isolated from houseflies (*P* < 0.05).

### Detection Rates of Apramycin Resistance Gene

**FIGURE 2:**
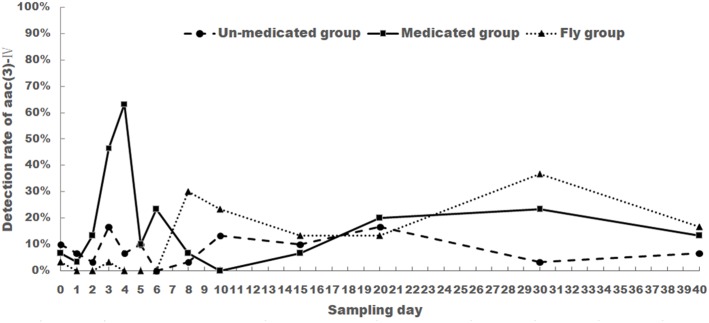
The changes of *aac(3)-IV* detection rate of *E. coli* isolated from chicken feces (medicated and un-medicated groups) and houseflies. Apramycin was administrated from Days 1 to 5 in their drinking water (0.5 mg/l) for the medicated group.

Apramycin resistance genes *aac(3)-IV* and *npmA* were screened for all 1170 *E. coli* isolates. *Aac(3)-IV* was detected in 32, 71, and 42 *E. coli* isolates from the un-medicated, medicated, and housefly groups, respectively. *npmA* gene was not detected in any samples from this study. The change of *aac(3)-IV* frequency is shown in **Figure 2**.

For the medicated group, *aac(3)-IV* detection rate was 6.67% before treatment (Day 0) and showed a steady increase from Day 1 (3.33%) to Day 4 (63.33%). Rates then decreased and fluctuated between 0 and 23.33% from Days 5 to 40. Noticeably, *aac(3)-IV* detection rates were still higher than Day 0. This rate held even 35 days after treatment (Day 40).

For the un-medicated group, *aac(3)-IV* detection rate showed no drastic change when compared to Day 0. Rates fluctuated between 3.33 and 16.67% for the entirety of the experiment.

For the housefly group, *aac(3)-IV* detection rate was low from Days 0 to 6 (0–3.33%), then increased and fluctuated between 13.33 and 36.67% from Days 8 to 40.

The *aac(3)-IV* detection rate was significantly different between medicated group and un-medicated group from days 3 to 4 (*P* < 0.05). No significant difference was found between un-medicated group and housefly group (*P* > 0.5).

### PFGE Typing of *aac(3)-IV*-Positive Strains

**FIGURE 3:**
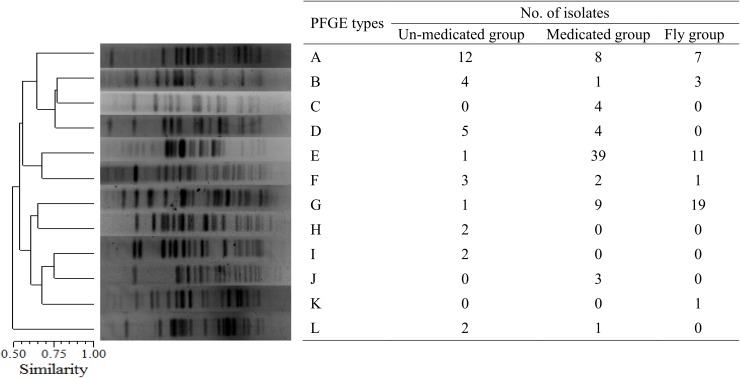
PFGE analysis of *aac(3)-IV*-positive *E. coli* isolates. A total of 145 *aac(3)-IV*-positive *E. coli* isolates from the un-medicated (*n* = 32), medicated (*n* = 71), and housefly groups (*n* = 42) were characterized into 12 PFGE types.

A total of 145 *aac(3)-IV*-positive *E. coli* isolates from the un-medicated (*n* = 32), medicated (*n* = 71), and housefly groups (*n* = 42) were analyzed using PFGE and 12 PFGE types were characterized (**Figure 3**). Among these, the three predominant PFGE types that emerged in the un-medicated group were types A (*n* = 12), B (*n* = 4), and D (*n* = 5). In the medicated group, the three major types were types A (*n* = 8), E (*n* = 39), and G (*n* = 9) and the housefly group were types A (*n* = 7), E (*n* = 11), and G (*n* = 19). PFGE types A, E, and G were the predominant types in both the medicated and housefly groups, suggesting houseflies play an important role in the spread of antibiotic-resistant *E. coli*.

### Characterization of Antimicrobial Resistance Phenotype and Genotype of *aac(3)-IV-*Positive Strains

Antimicrobial resistance profiles of the 12 *E. coli* isolates from each PFGE type are shown in **Table 2**. All tested isolates were multi-resistant, showing an antimicrobial-resistant phenotype to 10–18 antibiotics. Furthermore, all 12 isolates were co-resistant to the following antibiotics: ampicillin, tetracycline, doxycycline, ciprofloxacin, levofloxacin, gentamicin, and sulfamethoxazole/trimethoprim. They showed sensitivity to piperacillin/tazobactam, imipenem, meropenem, and amikacin. The number of isolates resistant to other antimicrobials ranged from 4 to 11 (**Table 2**).

**Table 2:** Antimicrobial resistance profile of *aac(3)-IV*-positive *E. coli* isolates of different PFGE types.

PFGE type	Resistance phenotype^a^	Resistance genotype
A	AMP, PRL, KZ, CTX, CRO, AMC, SAM, ATM, TE, DO, CIP, LEV, CN, SXT, C	*bla*_OXA_, *bla*_CTX-M-group 9_, *tetA, SulI*, *SulII*, *aac(6′)-Ib*, *ant(3^′′^)-Ia*, *aac(3)-IV*, *cmlA*, *intI*
B	AMP, PRL, KZ, CTX, CRO, SAM, TE, DO, CIP, LEV, CN, SXT, C, FFC	*bla*_TEM_, *bla*_CTX-M-group 9_, *tetA, SulII*, *ant(3^′′^)-Ia*, *aac(3)-IV*, *floR*, *cmlA*, *intI*
C	AMP, PRL, KZ, CAZ, CTX, CRO, FEP, SAM, ATM, TE, DO, CIP, LEV, CN, SXT, C, FFC	*bla*_TEM_, *bla*_CTX-M-group 1_, *bla*_CTX-M-group 9_, *tetA*, *SulI*, *SulII*, *aac(3)-IV*, *floR*, *intI*
D	AMP, SAM, TE, DO, CIP, LEV, CN, SXT, C, FFC	*bla*_OXA_, *tetA*, *SulI*, *SulII*, *aac(6′)-Ib*, *ant(3^′′^)-Ia*, *aac(3)-IV*, *floR*, *cmlA*, *intI*
E	AMP, PRL, KZ, CAZ, CTX, CRO, FEP, AMC, SAM, ATM, TE, DO, CIP, LEV, CN, SXT, C, FFC	*bla*_TEM_, *bla*_OXA_, *bla*_CTX-M-group 1_, *SulI*, *SulII*, *aac(3)-IIa*, *aac(6′)-Ib*, *ant(3^′′^)-Ia*, *aac(3)-IV*, *floR*, *cmlA*, *intI*
F	AMP, PRL, AMC, SAM, TE, DO, CIP, LEV, CN, SXT, C, FFC	*bla*_TEM_, *bla*_OXA_, *tetA*, *SulI*, *SulII*, *aac(3)-IIa*, *aac(6′)-Ib*, *ant(3^′′^)-Ia*, *aac(3)-IV*, *floR*, *cmlA*, *intI*
G	AMP, PRL, KZ, CAZ, CTX, CRO, FEP, AMC, SAM, ATM, TE, DO, CIP, LEV, CN, SXT	*bla*_TEM_, *bla*_OXA_, *bla*_CTX-M-group 1_, *tetA*, *SulI*, *SulII*, *aac(6′)-Ib*, *aac(3)-IV*, *intI*
H	AMP, PRL, KZ, CTX, CRO, FEP, SAM, ATM, TE, DO, CIP, LEV, CN, SXT, C, FFC	*bla*_CTX-M-group 9_, *SulII*, *aac(3)-IV*, *floR*
I	AMP, PRL, KZ, CTX, CRO, ATM, TE, DO, CIP, LEV, CN, SXT, C, FFC	*bla*_CTX-M-group 9_, *tetA*, *SulI*, *SulII*, *ant(3^′′^)-Ia*, *aac(3)-IV*, *floR*, *intI*
J	AMP, PRL, KZ, CTX, CRO, FEP, ATM, TE, DO, CIP, LEV, CN, SXT, C, FFC	*bla*_CTX-M-group 9_, *tetA*, *SulI*, *SulII*, *ant(3^′′^)-Ia*, *aac(3)-IV*, *floR*, *intI*
K	AMP, PRL, KZ, CTX, CRO, SAM, TE, DO, CIP, LEV, CN, SXT, C, FFC	*bla*_OXA_, *bla*_CTX-M-group 9_, *tetA*, *SulI*, *SulII*, *aac(6′)-Ib*, *ant(3^′′^)-Ia*, *aac(3)-IV*, *floR*, *cmlA*, *intI*
L	AMP, PRL, KZ, CAZ, CTX, CRO, FEP, SAM, ATM, TE, DO, CIP, LEV, CN, SXT, C, FFC	*bla*_CTX-M-group 1_, *bla*_CTX-M-group 9_, *tetA*, *SulII*, *aac(3)-IV*, *floR*, *intI*

*^a^AMP, ampicillin; PRL, piperacillin; KZ, cefazolin; CAZ, ceftazidime; CTX, cefotaxime; CRO, ceftriaxone; FEP, cefepime; AMC, amoxicillin/clavulanic acid; SAM, ampicillin/sulbactam; ATM, aztreonam; TE, tetracycline; DO, doxycycline; CIP, ciprofloxacin; LEV, levofloxacin; CN, gentamicin; SXT, sulfamethoxazole/trimethoprim; C, chloramphenicol; FFC, florfenicol.*

Resistance gene screening results showed multiple resistance genes co-existed in all 12 different *E. coli* isolates from each PFGE type (**Table 2**). The isolates among the 12 different PFGE types harboring resistance genes other than *aac(3)-IV* are shown in **Table 2**. Remarkably, 10 isolates harbored at least one ESBL genes (*bla*_CTX-M-group 1or9_). Moreover, among the 12 isolates, 11 were positive for the type I integrase gene *intI*.

## Discussion

Increasing attention has been paid to verify whether the extensive uses of antibiotics in food animals poses a risk to human health. Studies regarding the association between antibiotic administration and the development and persistence of resistant bacteria may provide guidance for more accurate antibiotic usage in animal husbandry.

Previous studies have suggested that apramycin administration can promote resistance *E. coli* isolated from swine (Mathew et al., 2003; Jensen et al., 2006). However, the influence of apramycin administration on *E. coli* resistance in chicken has not yet been reported. In this study, we demonstrated that the use of apramycin could facilitate *E. col I* resistance from the first day after administration to 1 day after cessation. Apramycin MIC90 dropped to a relatively low level 3 days after cessation, but increased again from Days 20 to 40 after cessation. Some studies have investigated the influence of other antibiotics on resistance changes of *E. coli* isolated from different farm animals (Smith et al., 2007; Martins da Costa et al., 2011; Sato et al., 2014). These previous studies have also demonstrated that antimicrobials caused selective pressure and resulted in increased resistance to bacteria originating from animals.

Noticeably, a high MIC90 was persistent even after stopping antibiotic treatment in the medicated group (Days 20–40). This value was higher than prior to antibiotic treatment, results that have also been found in a separate study (Smith et al., 2007). These findings could be due to the clonal dissemination of resistant strains and the capacity of *E. coli* to exchange resistance genes (da Costa et al., 2009). One of the potential reasons could be due to the dissemination of resistant strains by flies. Because according to the results of MIC90 of the flies group (**Figure 1**), the MIC90 values remained at a high level (256–1024 mg/ml) from days 20 to 40 in the housefly group.

MIC90 in the un-medicated group also increased at Day 3 and again from Days 10 to 20. This change in antibiotic resistance has also been observed in other studies featuring no antimicrobial treatment (Diarra et al., 2007; da Costa et al., 2009). These findings might be due to the influence of resistant strains in the farm environment and animal feed on microbial composition in the chicken gut (Apajalahti et al., 2004; Martins da Costa et al., 2011). We also hypothesized that the change of resistant phenotype of the un-medicated group was due to the spread of the resistant strains from the medicated group to un-medicated group through environmental factors (e.g., air, dust, mice, and flies). There are two reasons for this: first, compared with medicated group, the increase of MIC90 values of the un-medicated group was relatively delayed. Second, the trend of drug-resistant phenotype of the un-medicated group and housefly group was very similar, which suggested the resistant strains might be spread from the medicated group to un-medicated group by houseflies.

Furthermore, the influence of antimicrobial administration on resistance phenotype and genotype of *E. coli* isolated from houseflies captured from a poultry farm was investigated for the first time. Our study found that apramycin administration also promoted resistance of *E. coli* isolated from houseflies. However, the change of apramycin resistance in *E. coli* isolated from houseflies group was not as synchronous as that seen in the medicated group. To this end, MIC90 values rose from Days 2 to 6 (except for Day 5) in the medicated group, but remained at a low level (8–16 μg/ml) in the housefly group. Furthermore, while MIC90 values dropped from Days 8 to 15 in the medicated group, values rose above 256 μg/ml in the housefly group.

Pulsed-field gel electrophoresis analysis of *aac(3)-IV*-positive *E. coli* isolates indicated that the same strains were present in both fecal samples and houseflies. Furthermore, the predominant three PFGE types in the medicated group (A, E, and G) were also the predominant three PFGE types in the housefly group. This suggests that houseflies are transmission vehicles from chicken feces for resistant bacterial strains. Therefore, as the use of antimicrobials increases the presence of resistant strains in food producing animals, it will also likely increase the potential for further dissemination by houseflies to the public. Similar results have been found in pig farms, as *E. coli* isolates from flies and pigs showed the same resistance phenotype, genes, and PFGE profiles (Literak et al., 2009).

Resistance profiles of the *aac(3)-IV*-positive isolates of different PFGE types indicated multi-drug resistance was very common, which is consistent with other studies (da Costa et al., 2009; Zhang et al., 2009). Therefore, apramycin administration does not only cause selective effects on resistance itself, but also to other antimicrobials. Noticeably, among these apramycin-resistant isolates, the ESBL-producing strains were very common (10/12). More critically, some of these ESBL-producing strains also existed in houseflies. This would only increase their disseminating opportunity, posing a great potential risk to public health. Other studies have also shown that flies were capable of spreading ESBL-producing *E. coli* from poultry and cattle (Usui et al., 2013; Blaak et al., 2014).

## Conclusion

Our study found that apramycin administration increased the occurrence of *aac(3)-IV*-resistant isolates from chicken feces and houseflies. Moreover, houseflies transmitted resistant bacteria from chicken feces, thus increasing the potential risk of spreading these multi-resistant isolates to the public. Critical management strategies of antimicrobial usage in animal husbandry and pest control should be undertaken to better control and reduce this risk.

## Author Contributions

AZ and HW designed the study. ZG, YY, and YL carried out the sampling work. ZG, HT, and DL performed the experiments. AZ, CX, and CL analyzed the data. AZ and ZG drafted the manuscript. All authors have read and approved the final manuscript.

## Conflict of Interest Statement

The authors declare that the research was conducted in the absence of any commercial or financial relationships that could be construed as a potential conflict of interest.
